# Metal Ion Release from PEO-Coated Ti6Al4V DMLS Alloy for Orthopedic Implants

**DOI:** 10.3390/jfb16100362

**Published:** 2025-09-28

**Authors:** Shaghayegh Javadi, Laura Castro, Raúl Arrabal, Endzhe Matykina

**Affiliations:** 1 Departamento de Ingeniería Química y de Materiales, Facultad de Ciencias Químicas, Universidad Complutense de Madrid, 28040 Madrid, Spain; shjavadi@ucm.es (S.J.); rarrabal@ucm.es (R.A.); 2 Unidad Asociada al ICTP, IQM (CSIC), Grupo de Síntesis Orgánica y Bioevaluación, Instituto Pluridisciplinar (UCM), Paseo de Juan XXIII 1, 28040 Madrid, Spain

**Keywords:** titanium alloy, implant, additive manufacturing, plasma electrolytic oxidation, corrosion, metal release

## Abstract

This study investigates the influence of plasma electrolytic oxidation (PEO) on corrosion resistance of Ti6Al4V alloys produced by direct metal laser sintering (DMLS) for orthopedic implants. PEO (300 s) and flash-PEO (60 s) coatings containing Si, Ca, P, Mg and Zn were applied on both DMLS and wrought Ti6Al4V alloys. Samples, coated and uncoated, were characterized for microstructure, morphology and composition. Electrochemical behaviour was assessed by potentiodynamic polarization (PDP) and electrochemical impedance spectroscopy (EIS) in simulated body fluid (SBF) at 37 °C. Ion release was quantified by inductively coupled plasma optical emission spectroscopy (ICP-OES). DMLS alloy was more passive than wrought Ti6Al4V, releasing ~60% less Ti and ~25% less Al, but ~900% more V. For both alloys, correlation of corrosion current and ion release indicated that 98–99% of oxidized Ti remained in the passive layer. Flash-PEO produced uniform porous coatings composed of anatase and rutile with ~50% amorphous phase, while PEO yielded heterogeneous layers due to soft sparking. In both cases, coatings were the main source of ions. For the DMLS alloy, the best protection was afforded by flash-PEO, releasing 0.01 μg cm^−2^ d^−1^ Ti, 26 μg cm^−2^ d^−1^ Al, and 0.25 μg cm^−2^ d^−1^ V over 30 days.

## 1. Introduction

Titanium alloys, particularly Ti6Al4V, have long been valued for their exceptional strength-to-weight ratio, corrosion resistance, and biocompatibility. These properties make them indispensable in industries where performance and longevity are critical, especially in biomedical applications. The advent of additive manufacturing (AM), however, has revolutionized the processing of these alloys, enabling the fabrication of complex geometries that were previously unattainable through conventional manufacturing methods [1,2,3]. Techniques such as Selective Laser Melting (SLM) and Direct Metal Laser Sintering (DMLS) have facilitated the production of intricately tailored components for orthopedic implants and maxillofacial prostheses [4,5].

DMLS offers significant advantages in terms of material efficiency, design flexibility, and mechanical properties. However, the unique microstructural characteristics of AM Ti6Al4V alloys pose challenges, particularly regarding their corrosion resistance in aggressive environments [5]. A defining feature of AM-fabricated Ti6Al4V alloys is their fine-grained microstructure and the presence of a non-equilibrium martensitic α′ phase, which is a direct consequence of the rapid cooling rates inherent to AM processes. While this microstructure enhances mechanical properties such as hardness and tensile strength, it may also adversely affect the corrosion resistance of biomedical implants that interact with bodily fluids. Consequently, improving the corrosion resistance of AM-produced Ti6Al4V has become a research priority [5,6]. In particular, ceramic coatings have been shown to promote the formation of a more stable passive oxide layer and to increase surface bioactivity [7,8].

Surface modification using Plasma Electrolytic Oxidation (PEO) is a versatile strategy for enhancing the properties of AM titanium alloys. PEO relies on repeated microdischarges that occur when the applied voltage exceeds the dielectric breakdown strength of the growing oxide layer. These transient plasma discharges create localized regions of extremely high temperature (4000–12,000 K) and electron density, within which substrate material evaporates and oxidizes. The oxide produced in the plasma rapidly cools and solidifies, becoming incorporated into the coating structure. Each microdischarge consumes substrate at its site while simultaneously generating new oxide that grows both inward at the substrate–oxide interface and outward at the coating surface. Because the discharges recur across the surface in cascades, the coating is continuously restructured, leading to a porous but adherent ceramic with 1–50 µm thickness. This coating not only enhances corrosion resistance but also improves bioactivity, facilitating implant–cell interactions [5,6]. The effectiveness of PEO coatings against corrosion is highly dependent on process parameters, including electrolyte composition, applied voltage, and processing duration. Studies have shown that incorporating elements such as silicon, aluminum, and zirconium into PEO coatings further enhances corrosion resistance [7,8,9,10].

Recent research has provided valuable insights into the influence of PEO on the corrosion behavior of AM titanium alloys. Liu et al. demonstrated that SLM-Ti6Al4V implants exhibit narrower passivation behavior and increased susceptibility to corrosion compared to conventionally manufactured Ti6Al4V, likely due to their dominant α′ martensitic microstructure and reduced β phase [11]. More recently, Mora-Sanchez et al. observed that, regardless of the heat treatment and building orientation, the corrosion performance of AM Ti6Al4V alloy in 3.5 wt.% NaCl was inferior to that of the wrought alloy, suggesting that longer heat treatments producing larger β-phase grains might be beneficial for AM alloys [12]. The same authors explored the potential of short-time PEO treatments (flash-PEO), which create ultra-thin (3–10 μm) Ca- and P-containing coatings. Their findings revealed that even coatings as thin as 3 μm significantly improved the corrosion resistance of AM alloys, which exhibited faster coating growth due to their fine α-lamellar microstructure [5].

This study aims to bridge the knowledge gap with respect to the long-term corrosion resistance of AM Ti6Al4V and the effect of protective PEO coatings enriched in multiple bioactive elements on the release of potentially cytotoxic metal ions, compared to a reference conventionally manufactured Ti6Al4V. The corrosion behavior of heat-treated DMLS Ti6Al4V, with and without PEO coatings containing Ca, P, Zn, Si, and Mg, is investigated during a 30-day immersion in simulated body fluid (SBF) at 37 °C. Characterization of the oxide coatings and the AM substrate microstructure, electrochemical impedance spectroscopy (EIS), potentiodynamic polarization (PDP), and analysis of Ti, Al, and V ion content in the SBF after corrosion are used to elucidate the corrosion mechanism of AM-produced alloy.

## 2. Materials and Methods

### 2.1. Ti6Al4V Substrates

The reference material used was a wrought, conventionally processed (i.e., hot-rolled, mill-annealed) 0.5 mm thick Ti6Al4V sheet. The AM Ti6Al4V specimens were produced using DMLS method of additive manufacturing by Fundación Idonial (Asturias, Spain) with an EOS M280 system. Information regarding powder feedstock and printing pattern was previously reported elsewhere [5].

Post-processing of AM alloy included a stress-relief treatment at 300 °C for 2 h, followed by furnace cooling. To transform the α′ acicular microstructure into an α + β lamellar structure, an additional heat treatment at 750 °C for 3.5 h was applied. Specimens (30 × 10 × 3 mm) were extracted in the XY direction.

### 2.2. Plasma Electrolyte Oxidation

Before PEO treatment, specimens were ground with SiC sandpapers up to P1200 and chemically etched for 180 s in a modified Keller solution (95.5 mL H_2_O, 2.5 mL HNO_3_, 1.5 mL HCl, and 0.5 mL HF). Grinding was done to eliminate the oxide scale formed after the heat treatment, removing approximately 20 µm of material. Etching removes the natural oxide layer and surface contaminants, exposing fresh metal and increasing reactivity. It also helps to trace the microstructural features before and after PEO and corrosion testing. The samples were then attached to a copper wire for electrical contact, and insulated with No. 45 stopping-off Lacquer (MacDermid Enthone, Waterbury, CT, USA), leaving a working area in each side of total 4 cm^2^.

The electrolyte was an aqueous solution containing Ca, P, Zn, Mg and Si as bioactive species, with the following composition: Ca(CH_3_COO)_2_·10H_2_O 15.4 g/L, NaH_2_PO_4_·2H_2_O 3.9 g/L, Na_2_EDTA·2H_2_O 20.5 g/L, ZnO 2 g/L, Na_2_SiO_3_·5H_2_O 3 g/L, MgSO_4_ 3 g/L, pH 5.0. The PEO treatment was carried out with an AC square voltage signal with 490 V positive pulse and 30 V negative pulse (V_RMS_ = 347 V) at 300 Hz and 50% duty cycle, using a 2 kW regulated power supply (EAC-S2000, ET Systems Electronic, Altlussheim, Germany). The initial rise of the voltage was controlled by a 60 s ramp. The RMS current density was limited to a maximum of 300 mA·cm^−2^. The duration of the experiment was 60 s (flash-PEO) and 300 s (PEO) for both reference and AM alloys. The process was performed in a 2 L, water-jacketed glass cell thermostated at 20 °C. A 316L stainless steel mesh cylinder served as the counter electrode.

Coating thickness was measured using an eddy-current meter (ISOSCOPE FMP10, Fischer Instruments, Barcelona, Spain) with an FTA3.3H probe, averaging 10 measurements.

### 2.3. Material Characterization

Surface topography characterization was conducted using an Infinite Focus SL system (Alicona GmbH, Graz, Austria). Both 2D and 3D surface reconstructions were performed, and surface topography data (S_10z_ and S_a_) were analyzed with IF-Measure Suite v5.3 software.

The surface and cross-sectional morphology of the coatings was examined using a JEOL JSM-6400 scanning electron microscope (SEM) (JEOL Ltd., Akishima, Japan) with an Oxford Link energy-dispersive X-ray spectroscopy (EDS) facility. Pore size and population density were analyzed from plan-view SEM images using ImageJ v1.5.3t software.

For phase analysis, X-ray diffraction (XRD) was performed with a Bruker D8 ADVANCE A25 diffractometer (Billerica, MA, USA) fitted with a CuKα radiation source (λ = 0.154056 nm). Scanning was carried out between 10° and 80° 2θ at 0.05°/s, and the data were analyzed using Panalytical’s X’Pert HighScore v5.1 software with the ICDD PDF5+ database.

Surface wettability was evaluated using the FTA1000 Drop Shape Analysis System of First Ten Angstroms (Newark, CA, USA). Sessile drop measurements were initiated in a time interval of 25 s after contact, capturing 50 frames. Three drops were used to obtain an average contact angle.

### 2.4. Corrosion Testing

For corrosion testing, the exposed area of bare and coated conventional and AM specimens was delimited to 0.95 cm^2^ using an O-ring cell.

SBF of the following composition was used as an electrolyte, g/L: NaCl 8.035, NaHCO_3_ 0.355, KCl 0.225, K_2_HPO_4_·3H_2_O 0.231, MgCl_2_·6H_2_O 0.311, CaCl_2_ 0.292, Na_2_SO_4_ 0.072. This formulation was based on c-SBF reported by Oyane et al. [13] but stripped of TRIS buffer. Buffers like TRIS and HEPES have been reported to alter the in vitro corrosion performance of biomaterials [14,15].

Electrochemical tests were conducted using a Gamry Interface1010E Potentiostat/Galvanostat/ZRA (Gamry Instruments, Warminster, PA, USA) with an Ag/AgCl reference electrode and a 1 cm^2^ Pt counter electrode. A double-walled electrochemical cell connected to a thermostated water bath maintained the electrolyte temperature at 37 ± 0.5 °C. At least two specimens per condition were tested for repeatability.

The testing sequence included 1 h of open-circuit potential (OCP) stabilization, followed sequentially by EIS and PDP measurements. Subsequently, only EIS measurements were carried out periodically until the end of immersion period of 30 d. EIS was performed with a 10 mV sinusoidal signal relative to OCP across a frequency range of 10^5^ to 0.01 Hz. Impedance spectra were fitted using ZView 3.1c software, ensuring chi-squared values below 0.01 and parameter errors under 5–10%. Polarization curves were recorded between −0.15 V and 3 V relative to OCP at a scan rate of 0.5 mV/s.

### 2.5. Metal Ion Release

After 30 days of immersion in SBF, the Ti^4+^, V^5+^ and Al^3+^ ion release was analyzed in all solutions by inductively coupled plasma optical emission spectroscopy (ICP-OES), using a Perkin Elmer Avio 220 Max instrument equipped with a cyclonic spray chamber and operated at RF power of 1500 W, plasma gas flow of 10 L/min, auxiliary gas flow of 2.0 L/min, nebulizer flow of 0.7 L/min, and pump flow rate of 1 mL/min. Argon was used as the plasma-maintaining carrier gas. The blank of SBF was included in the batch measurements as a reference. Two samples per condition were used for repeatability. Each measurement was performed in triplicate with relative standard deviation (RSD) < 3%.

## 3. Results

### 3.1. Substrate Characterization

**Figure 1:**
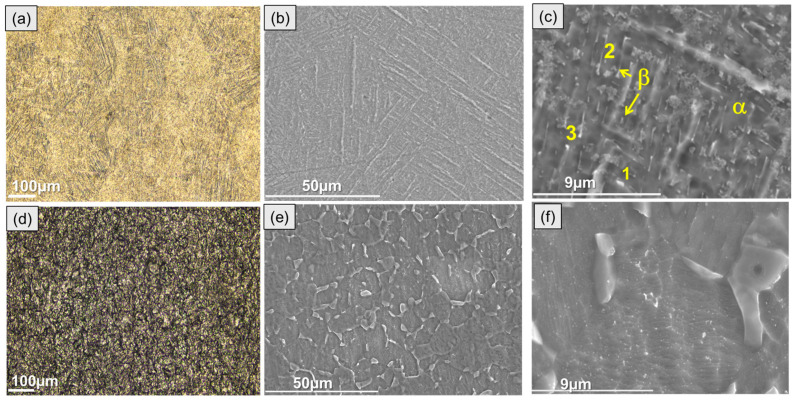
Optical (**a**,**d**) and secondary electron micrographs (**b**,**c**,**e**,**f**) of ground and etched DMLS Ti6Al4V (**a**–**c**) and wrought Grade 5 (**d**–**f**) alloys.

Figure 1 shows the scanning electron microscope (SEM) images of the microstructure of conventional and additively manufactured titanium alloys after metallographic surface preparation. Typically, the as-built DMLS Ti6Al4V microstructure is composed of α/α′ acicular grains, i.e., mostly martensite, as described elsewhere [12]. Following heat treatment, the AM alloy acquires a fine lamellar microstructure, with a distinct difference between the α and β phases (Figure 1a,c). The β-phase presents a brighter contrast due to its greater vanadium content (Table 1), which is a β-phase stabilizer.

The wrought alloy microstructure was mostly composed of coarse-grained equiaxed α-phase with angular β-phase located at the α-grain boundaries (Figure 1d–f).

### 3.2. Coating Growth and Characterization

The PEO electrical regime parameters used in the present work were previously established in [16] for wrought c.p. Ti Grade I alloy, ensuring sustainable, uniformly distributed microdischarges, a uniform coating thickness and incorporation of bioactive elements from the electrolyte into the coating. In the present work, we explore the effect of these electrical parameters and the treatment time on the coatings formed on wrought and AM Ti6Al4V alloys. It should be noted that extended treatments are often counterproductive for the quality of the coating and lead to high energy consumption. For this reason, the possibility of satisfactory incorporation of bioactive elements during 60 s and 300 s treatments is investigated here.

**Figure 2:**
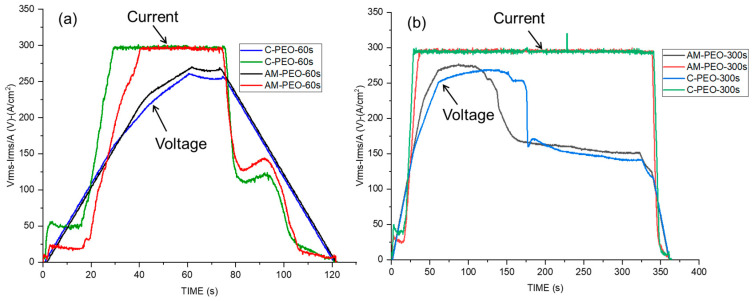
Voltage and current Response Vs. Time during PEO treatments of conventional (C) and DMLS (AM) substrates: (**a**) flash-PEO, (**b**) PEO (300 s).

Figure 2a,b present the variation in the root means square values of voltage (V_RMS_) and current density (I_RMS_) with time during the PEO processing of wrought and AM alloys for 60 and 300 s.

The long and short treatment curves for both AM and conventional alloys reveal that the I_RMS_ remains constant (~25 and ~50 mA/cm^2^, respectively) throughout the first 20 s of the process, whereas the V_RMS_ continues to rise, indicating the oxide layer thickness increase. Evidently, oxidation is easier in AM alloy, since it requires lower current density to reach ~75 V. After ~20 s, the I_RMS_ of both conventional and additive alloys rapidly reached the set limit of 300 mA·cm^−2^. This rise was associated with the increase in electronic conductivity of the oxide and subsequent dielectric breakdown and appearance of plasma microdischarges. Visually, the first sparks appeared earlier on the conventional alloy (at ~22 s) than on the AM alloy (~25 s).

During the long PEO process, the voltage declined to ~150 V. The transition occurred earlier on AM than on wrought alloy, after ~100 s and ~150 s, respectively. Visually, this resulted in smaller, more uniform and silent microdischarges initiated at the edges of the exposed area, a phenomenon known as “soft sparking” [17], whereas everywhere else the “hard” (i.e., acoustic-emission accompanied) sparking essentially ceased.

**Figure 3:**
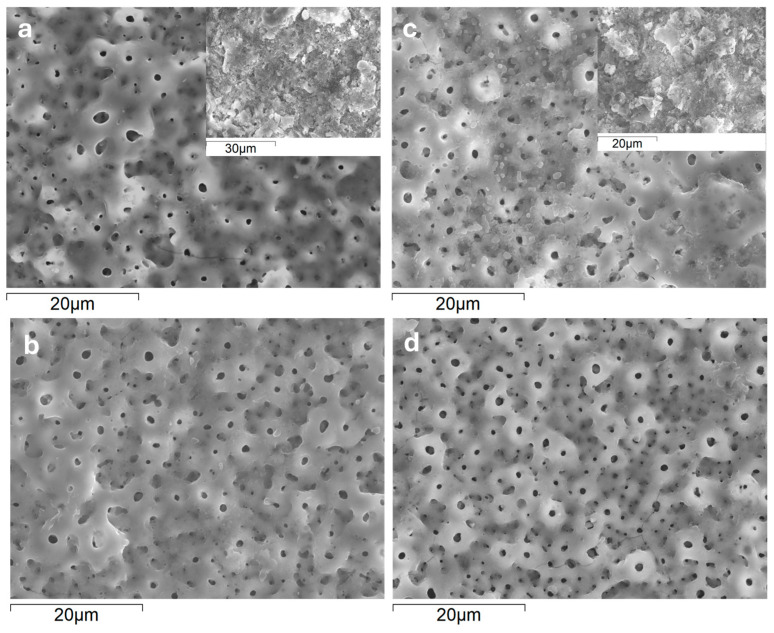
Surface morphology of coatings formed on wrought (**a**,**b**) and AM (**c**,**d**) alloys during 300 s (**a**,**c**) and 60 s (**b**,**d**). Insets in (**a**,**c**) show the morphology in the areas affected by soft sparking.

The SEM images *(*Figure 3*)* and the plan-view area EDS analysis of the coatings (Table 2) disclosed the presence of Ca, P, Si, Mg, and Zn originating from the electrolyte solution. The calculated Ca/P ratios, about 0.9, indicate the likely formation of calcium phosphate phases, which are known for their ability to promote tissue regeneration [16]. In addition to oxygen and titanium, associated with the formation of TiO_2_, EDS analysis revealed the presence of Na derived from the electrolyte, as well as Al and V originating from the substrate. Prolongation of the treatment time from 60 s to 300 s did not notably affect the surface levels of the substrate- and the electrolyte-derived species. This may be related to the fact that from 100–150 s onwards, “hard” sparking was replaced by “soft” sparking at the edges of the samples (Supplementary Figure S1).

On the other hand, porosity estimated from image analysis (Table 3) revealed a clear correlation with the PEO treatment time. In both alloys, longer treatment produced an increase in average pore size from <1 um up to ~3 µm, and a decrease in pore population density by 2–4 times. As a result, a small increase in porosity, by 4–6%, was observed, without significant differences between the wrought and AM alloys. Importantly, “soft” sparking resulted in some local heterogeneity (insets, Figure 3a,c), producing virtually pore-free surface at locations where it took place. In both alloys, Si and O predominated in such areas, suggesting formation mainly of SiO_2_ (Table 2).

**Figure 4:**
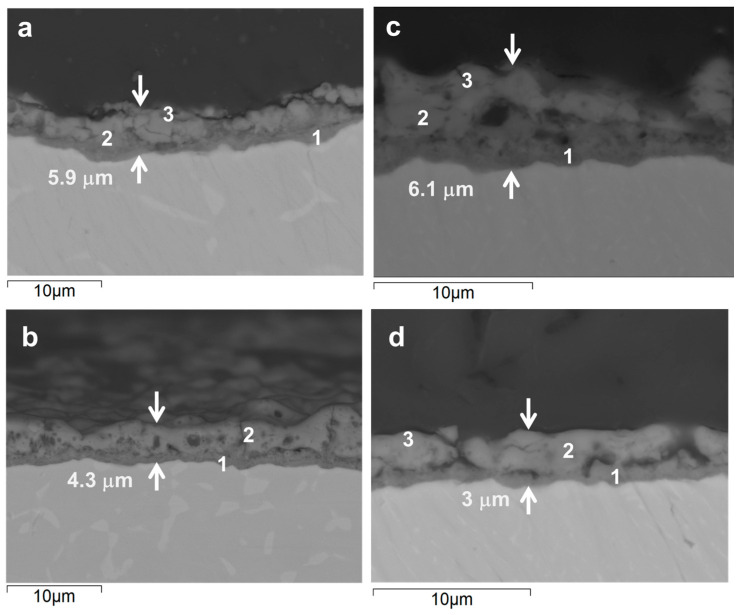
Cross-sectional backscattered electron micrographs of the coatings formed on wrought (**a**,**b**) and AM (**c**,**d**) alloys during 300 s (**a**,**c**) and 60 s (**b**,**d**).

Figure 4 shows backscattered electron images of cross-sections from coated Ti-6Al-4V alloys. These images highlight clear differences in the thickness and morphology of the resulting oxide layers. To probe the composition, EDS analysis was performed on specific regions, providing the information about the distribution of elements across the coating thickness (Table 4).

In the conventional Ti6Al4V alloy, a relatively thick oxide layer (~5.9 µm) was formed after 300 s of PEO treatment (Figure 4a). This layer had a distinct duplex structure, with a dense barrier layer (~0.75 µm) and a microcracked outer region. According to the EDS analysis, the inner layer was richer in Ti, Al and V compared with the outer one (Table 4). The outer region contained more P, Si, Ca, Mg and Zn (in descending order). On average, the electrolyte-derived species were incorporated at ~1.0–5.0 at.%. In comparison, after just 60 s of PEO (Figure 4b), the oxide layer was slightly thinner (4.3 µm), with micrometric pores discernable in the outer region. The EDS results for this stage indicated successful incorporation of the electroltye species into the coating at a similar or greater level than in 300 s PEO, with Ca content being particularly high (7.3 At.% Vs. 1.8 at.%). Notably, the V content was about twice as low for 60 s PEO (0.7 At.% Vs. 1.4 at.%).

The AM samples behaved similarly to the conventional ones under the same PEO conditions. After 300 s of treatment (Figure 4c), the oxide layer on the AM alloy was of similar total thickness (~6.1 µm) and barrier layer thickness, but with a more porous outer layer structure. The most notable finding of the EDS measurements taken across the coating thickness profile (points 1–3) was at least two times lower V content, with all else being at similar or slightly lower levels compared to the wrought alloy counterpart. After a flash 60 s treatment (Figure 4d), the oxide layer was at its thinnest (~3.0 µm) and featured a lateral continuity of pores in the inner region that appeared to originate from the bottle-neck of discharge channels. EDS analysis revealed a composition similar to that of the coated conventional counterpart, but with slightly greater oxygen content and lower levels of the other elements.

**Figure 5:**
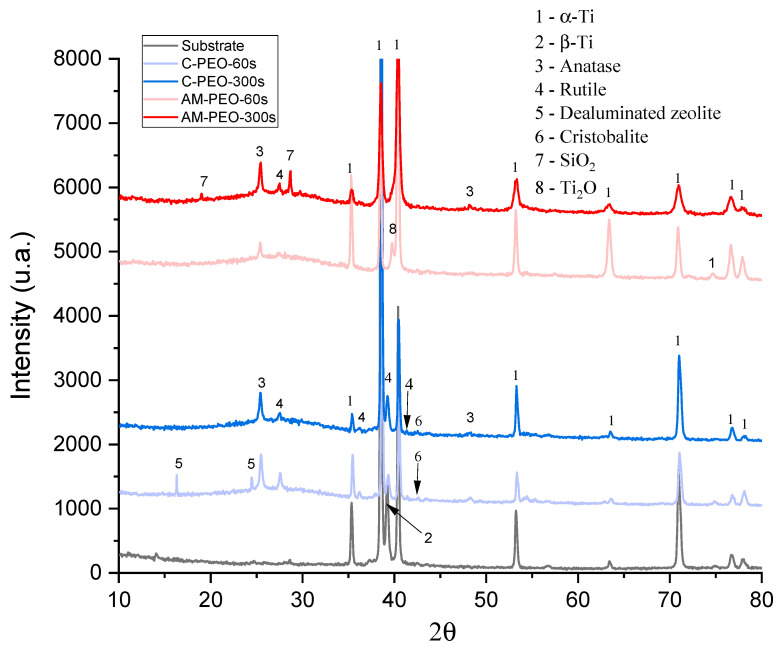
XRD patterns of flash PEO and PEO coatings on conventional (C) and DMLS (AM) substrates. Substrate pattern corresponds to conventional Grade 5 alloy.

XRD results of PEO- and flash-PEO-coated conventional and DMLS alloys are shown in Figure 5.

It is evident that coatings on wrought alloy develop a greater amount of rutile, which forms even in flash-PEO. The wrought alloy develops crystalline SiO_2_, dealuminated zeolite and cristobalite (ICDD PDF 00-045-0112 and ICDD PDF 00-004-0379, respectively) in flash-PEO coating, while in the AM alloy this phase is detected only after 300 s of PEO processing. Notably, a lower oxidation state of Ti is observed in AM alloy, according to the presence of Ti_2_O. Other observed diffraction peaks correspond to the hexagonal close-packed alpha phase of titanium. In all four coatings, a substantial amount of amorphous phase was observed (as indicated by the curved background between ~20–35° 2θ), which increased with PEO treatment time from 40.4% to 53.3% for wrought alloy, and from 56.4% to 60% for AM alloy.

### 3.3. Corrosion Behavior

#### 3.3.1. Open Circuit Potential and Potentiodynamic Polarization

**Figure 6:**
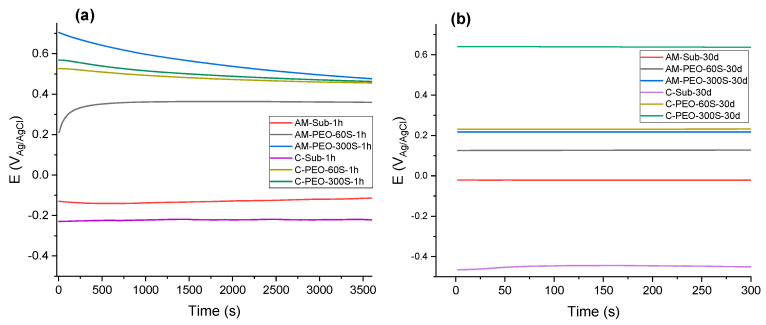
OCP of conventional (C) and DMLS (AM) substrates without and with flash-PEO and PEO (300 s) coatings: (**a**) after 1 h and (**b**) after 30 days of immersion in SBF.

The OCP, also known as the corrosion potential, characterizes the thermodynamic tendency of a material to corrode; more negative values correspond to a greater propensity for corrosion. The OCP values of the samples after 1 h and 30 days of immersion in SBF reflect their long-term electrochemical stability (Figure 6). The OCP values stabilized within 30 min of immersion, with a variation of <1 mV by 3600 s. All coated samples exhibited ~450–650 mV more noble OCPs than respective bare substrates. As the data indicate, most systems experienced a decrease in OCP values after 30 days, which can be attributed to the prolonged exposure to the corrosive environment affecting the coating structure and/or passive film thickness on the substrates.

The uncoated conventional titanium alloy, in particular, experienced the most significant drop in potential long-term, with the OCP decreasing from −0.22 V (after 1 h) to −0.44 V (after 30 days). The AM substrate, on the other hand, showed an increase in OCP, from −0.13 V to −0.02 V.

Among the PEO-coated samples, the PEO-coated conventional alloy showed the highest stability, with a positive shift in OCP from 0.47 V (after 1 h) to 0.66 V (after 30 days). The rest of the coated systems exhibited a decrease in OCP long-term; however, their values remained in the positive range.

**Figure 7:**
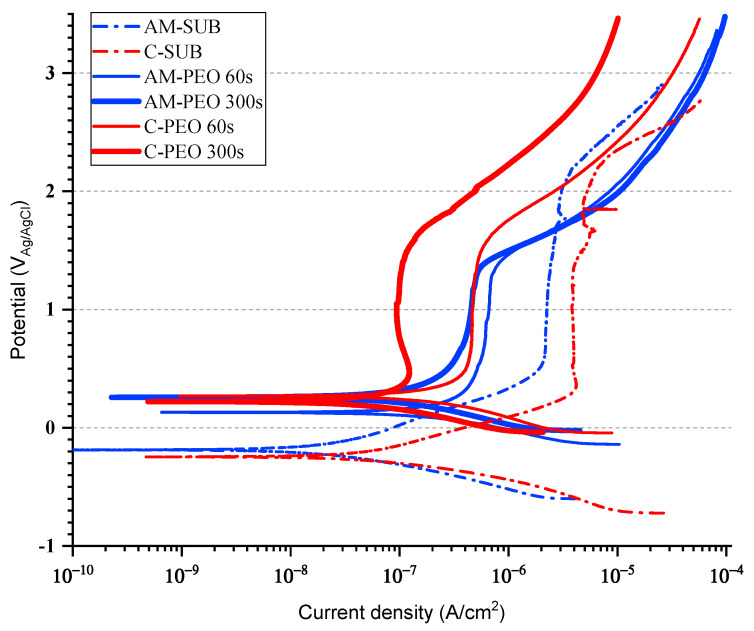
PDP curves of conventional (C) and DMLS (AM) substrates without and with flash-PEO and PEO (300 s) coatings after 1 h of immersion in SBF.

The results of potentiodynamic polarization (Figure 7) reveal that flash-PEO and PEO treatments significantly improve the corrosion resistance of both alloys. The untreated samples (AM-SUB and C-SUB) exhibited the highest corrosion current density (i_corr_), a parameter related to the corrosion rate or metal loss, with the AM alloy showing better performance (i_corr_ 0.05 μA/cm^2^) compared to the conventional one (i_corr_ 0.17 μA/cm^2^, Table 5). The latter value is common for bare Ti6A4Al in physiological media [18].

Interestingly, flash-PEO treatment slightly increased the i_corr_ from 0.17 μA/cm^2^ to 0.19 μA/cm^2^ and from 0.05 μA/cm^2^ to 0.16 μA/cm^2^ for the wrought and AM alloys, respectively. Only the 300 s PEO treatment of the AM alloy resulted in 50% lower i_corr_ compared with the bare substrate. The passive current densities for the coated systems, i_pass_ (the oxidizing current needed to maintain the thickness of a passive oxide film constant), decreased with increasing PEO treatment time, becoming ~2.5–25% of the original value. Both treatments had a greater passivating effect on conventional alloy than on the AM one.

As seen in the PDP curves, none of the materials showed any evidence of pitting corrosion. Instead, a transpassive behavior associated with O_2_ evolution was observed above ~2.2 V, ~1.4 V and ~1.6 V for bare substrates, flash-PEO and PEO coatings, respectively.

#### 3.3.2. Electrochemical Impedance Spectroscopy

**Figure 8:**
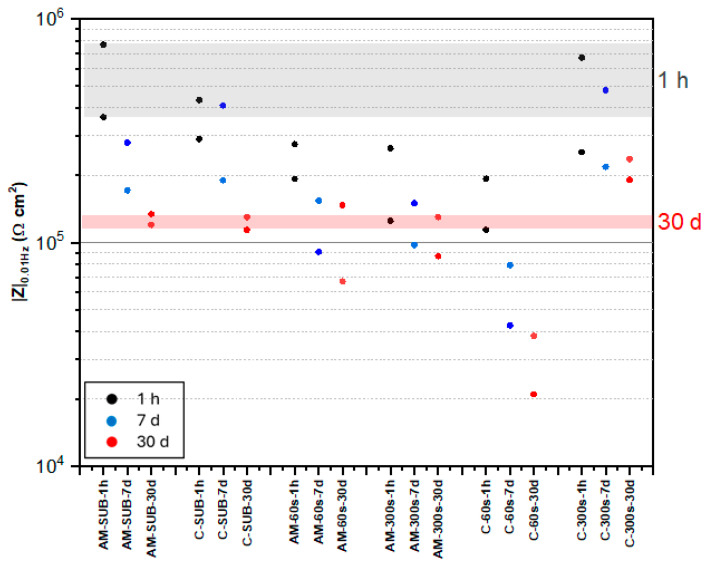
Scattered diagram of total impedance values at 0.01 Hz for bare and coated AM and conventional alloy substrates after 1 h, 7 days and 30 days of immersion in SBF. Shaded areas correspond to the reference |Z|_0.01Hz_ values for bare AM substrate after 1 h and 30 days of immersion.

Figure 8 shows the evolution of the total impedance modulus values at low frequency (0.01 Hz) over time for all studied systems. The impedance modulus represents the overall resistance of the material to the passage of alternating current. A steady decrease in |Z|_0.01Hz_ values by 3–4 times occurred in all cases. After 30 days of immersion, no significant difference was observed between the values for non-coated AM and wrought alloys. The PEO (300 s) coating on wrought alloy demonstrated the greatest |Z|_0.01Hz_ value long-term, which is in agreement with the lowest passive current density observed for this system (Figure 7). The flash-PEO coating on wrought alloy yielded the lowest |Z|_0.01Hz_ value long-term among all systems.

**Figure 9:**
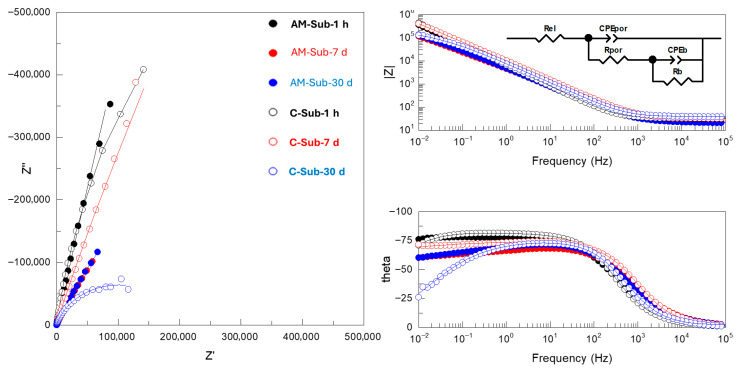
Nyquist and Bode plots of EIS spectra and the equivalent circuit used to fit EIS data for AM and conventional Ti6Al4V alloy substrates after 1 h, 7 days and 30 days immersion in SBF. Real, imaginary and total impedance units are Ω cm^−2^. Symbols and continuous lines correspond to the experimental and fitted data, respectively.

**Figure 10:**
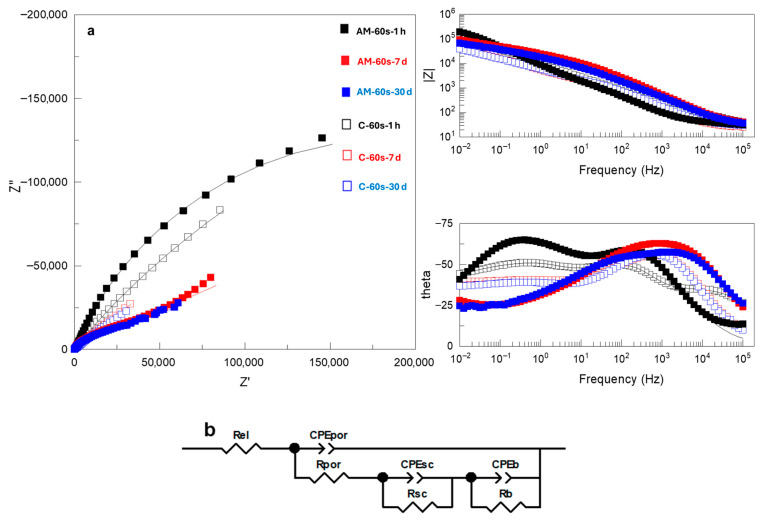
(**a**) Nyquist and Bode plots of EIS spectra and (**b**) equivalent circuits used to fit EIS data for flash PEO-coated AM and conventional Ti6Al4V alloys after 1 h, 7 days and 30 days immersion in SBF. Real, imaginary and total impedance units are Ω cm^−2^. Symbols and continuous lines correspond to the experimental and fitted data, respectively.

**Figure 11:**
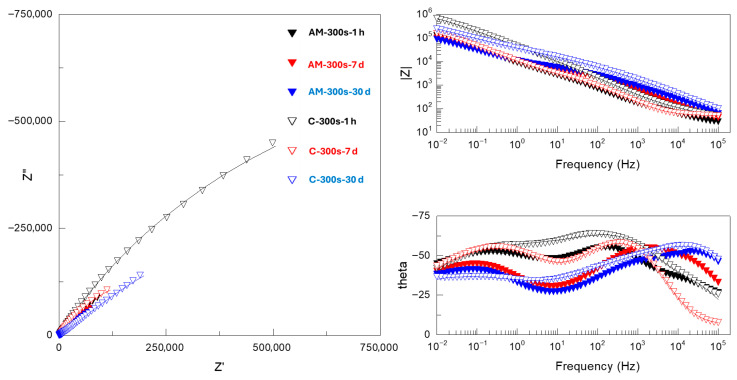
Nyquist and Bode plots of EIS spectra for PEO-coated (300 s) AM and conventional Ti6Al4V alloys after 1 h, 7 days and 30 days of immersion in SBF. Real, imaginary and total impedance units are Ω cm^−2^. Symbols and continuous lines correspond to the experimental and fitted data, respectively.

It is evident that |Z|_0.01Hz_ values cannot be directly correlated with the protective capacity of the coatings, since in nearly all systems both short- and long-term behavior do not match that observed during potentiodynamic polarization. The detailed analysis of Nyquist and Bode plots of the EIS data (Figure 9, Figure 10 and Figure 11 and Supplementary Tables S1–S3) provides further insight into the observed behavior. The equivalent electrical circuits (EEC) used to fit the experimental data employed constant phase elements (CPE) [18] instead of capacitance. CPE is used to reflect the defects and heterogeneities in the passive films, and coating/substrate interface when these do not behave as ideal capacitors.

Bare substrates disclosed a capacitive behavior (i.e., phase angle θ close to −90°, Figure 9) in the 10^2^–10^−2^ Hz range of frequencies, which is typical for Ti alloys in neutral saline media [12]. This range narrowed slightly for the wrought alloy following 30 days of immersion. The experimental data were adequately fitted to a two-time constant EEC (Figure 9, inset), which were ascribed to the response of the porous (R_por_/CPE_por_) and barrier (R_b_/CPE_b_) oxide film formed on different grains of the substrate. It is inferred that the pores, or defects, of the film are electrolyte-filled, given that the resistance values, R_por,_ are as low as the resistance of the electrolyte (Supplementary Table S1).

Flash-PEO-coated alloys (Figure 10a) disclosed three time constants (i.e., three maxima in the Bode plot of the phase angle). These correspond to the response of the porous region of the coating (R_por_/CPE_por_) at high frequencies, the space charge region (R_sc_/CPE_sc_) at intermediate-low frequencies and, lastly, the barrier layer of the coating (R_b_/CPE_b_). This response was fitted to the EEC (Figure 10b) used in our previous work [5] and commonly recommended for anodic films and PEO coatings on Ti alloys [19,20]. It is evident, particularly in the case of the AM alloy, that with immersion time the response of the inner and outer regions of the coating becomes less separated in time, as the pores of the outer region become blocked (R_por_ increases, Supplementary Table S2). In the case of flash-PEO on conventional alloys, the pores remain highly conductive, i.e., electrolyte-filled.

PEO-coated alloys (Figure 11) disclosed a three-time constant response similar to that of flash-PEO systems. Therefore, the experimental spectra were fitted using the same EEC (Figure 10b). A notable feature of both longer PEO and flash-PEO treatments at medium and low frequencies, i.e., across the barrier layer and the space-charge region, is the θ angles approaching 40–45° with immersion time and exponential factors *n* as low as 0.33 (Supplementary Tables S2 and S3). At medium frequencies, this may be attributed to the diffusion of species through the nanopores of the barrier layer. At low frequencies, it is likely due to the compositional heterogeneity caused by the incorporation of Al^3+^ and V^5+^ species into the barrier oxide from the substrate, which is in agreement with other reports [21].

**Figure 12:**
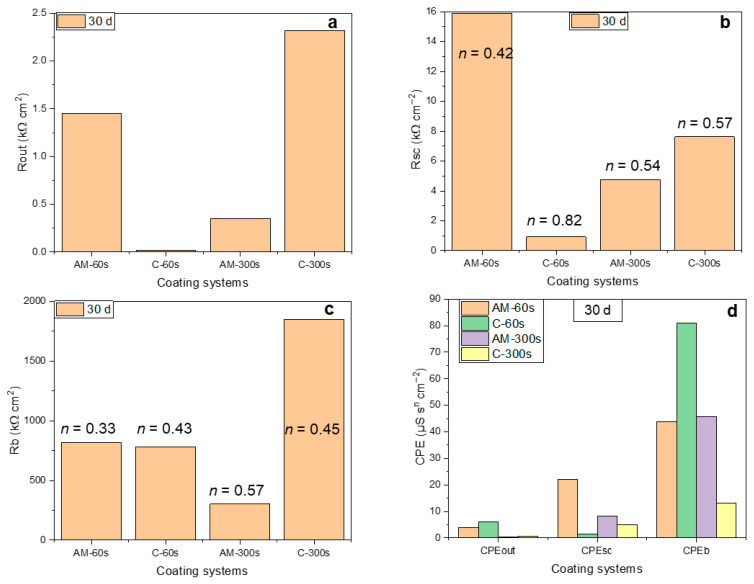
Selected parameters derived from fitting the EIS spectra: resistance (**a**–**c**) and CPE (**d**) values of the outer coating region (**a**), coating/substrate interface (**b**) and barrier layer (**c**) following 30 days of immersion.

Figure 12 compares the response of the three distinct regions of the coated systems, i.e., the porous and barrier layers of the coatings and the space-charge region, following 30 days of exposure. The presented values were obtained from the analysis of the EECs parameters (Supplementary Tables S2 and S3). The pores of the outer region of the flash-PEO coating on the AM alloy become clogged, while on the wrought alloy they remain fully permeable by the electrolyte (R_out_ ~2500 Ωcm^2^ vs. ~30 Ωcm^2^_,_ Figure 12a). For the PEO coating, the pore clogging is observed for the wrought alloy and is negligible for the AM one.

For both alloys, the resistance of the space-charge region in the barrier layer is 5–10 times greater than that of the outer region (Figure 12b). This region of the film is not defect-free, as indicated by the *n* values in the range of 0.54–0.75. The defects in the barrier layer are typically nano-voids [22,23] that can be permeated by the electrolyte and allow for the diffusion of cations and anions [24]. The space-charge region resistance was greatest for the flash-PEO coating on the AM alloy. The barrier layer resistance, R_b_, appears to dominate in all cases (~0.4–20 MΩcm^2^), being greatest for the PEO-coated conventional alloy (Figure 12c). Values of this order are typical for anodic barrier layers on Ti alloys [5,20]. In terms of capacitance (Figure 12d), the space-charge region of the coatings exhibits the CPE_sc_ values ranging within ~2–20 μSs^n^cm^−2^_,_ typical for semiconductive TiO_2_ [20], while the CPE_b_ values (~40–80 μSs^n^cm^−2^ in most of the coated systems) indicate the existence of mass transfer limitations through the coating material.

### 3.4. Coating Characterization After Corrosion

**Figure 13:**
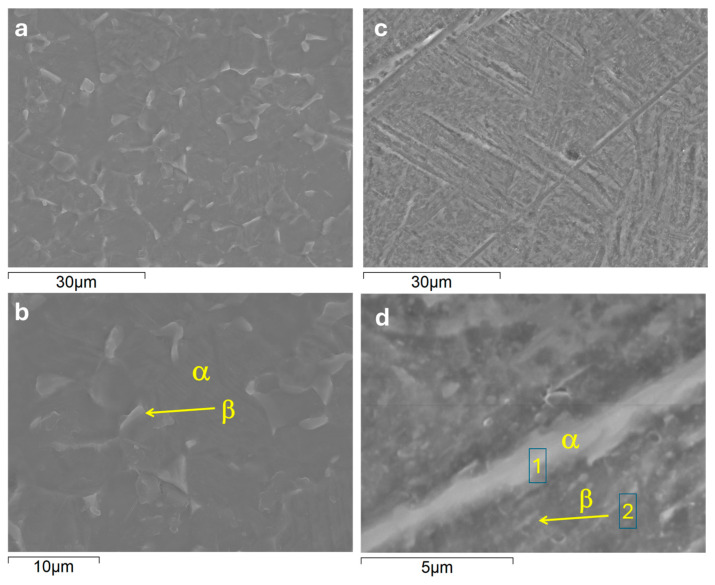
Surface morphology of wrought (**a**,**b**) and AM (**c**,**d**) alloys after 30 days of immersion in SBF.

**Figure 14:**
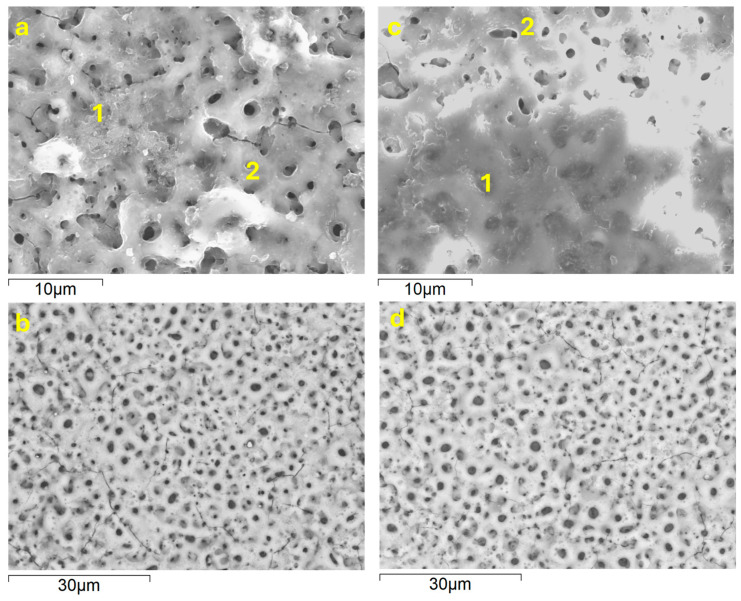
Surface morphology of PEO (**a**,**c**)- and flash-PEO (**b**,**d**)-coated wrought (**a**,**b**) and AM (**c**,**d**) alloys after 30 days of immersion and EIS testing in SBF. (**a**,**c**) correspond to secondary electron and (**b**,**d**) to backscattered electron imaging.

Figure 13 and Figure 14 show the SEM examination of AM and conventional alloys, without and with coatings, respectively, following 30 days of corrosion testing. It is evident that the bare substrate surface morphology has not changed compared with the initial state (Figure 1), showing no signs of uniform or localized degradation. Composition-wise, the surface became enriched in oxygen (Table 1), as expected, due to the growth of oxide films under the applied polarization.

Likewise, the morphologies of coated samples reveal no signs of corrosion damage. In the case of PEO coatings on both conventional and AM alloys, flaky deposits are seen (Figure 13a,c). For the PEO-coated AM alloy, the deposits appear to be more continuous and clogging the pores (Figure 13c). EDS analysis (Table 6) in both cases reveals an increase in Ca compared with the as-received state (Table 2), from 3.4 to 6.5 at.% and from 1.9 to 9 at.% for conventional and AM alloys, respectively. Ca/P ratios increased from 0.5 to 1.1 and from 0.31 to 1.1, respectively, indicating the precipitation of Ca-P compounds from SBF. Nevertheless, in both cases, the Ca/P ratios remained below those for apatite or hydroxyapatite, which may be due to the periodic interference of the applied polarization during EIS testing.

Notably, flash-PEO coatings did not induce the precipitation of Ca-P phases from SBF, as both Ca and P content decreased considerably (almost by 2 times in the case of Ca) post-immersion. Ca/P ratios went down from 1.25 to 0.96–0.98 in both alloys, i.e., these coatings promoted leaching of both Ca and P. Other bioactive elements that had been added to the coatings from the electrolyte during the PEO process exhibited variably lower levels, suggesting their release.

### 3.5. Roughness and Water Contact Angle

The roughness and wettability data presented in Table 7 were derived from the topographical images (Supplementary Figure S2) and water contact angle (WCA) measurements, respectively, acquired before and after 30 days of corrosion exposure.

The WCA values for both alloys decreased after 30 days of immersion, indicating that the alloy surface became more hydrophilic as a result of passivation. In the coated alloys, the general trend was a slight decrease in hydrophilicity, except the PEO-coated AM alloy, which exhibited a lower WCA. This behavior may be related to a greater amount of precipitates from SBF, consistent with the considerable increase in the S_a_ value and observed morphological changes (Figure 14c).

For the flash-PEO coatings, the decrease in hydrophilicity correlated with a decrease in roughness. This effect may be associated with the lixiviation of bioactive elements detected by EDS and the corresponding changes in surface chemistry, which could influence the chemisorption of water molecules.

### 3.6. Ion Release After 30 Days of Corrosion Exposure

**Figure 15:**
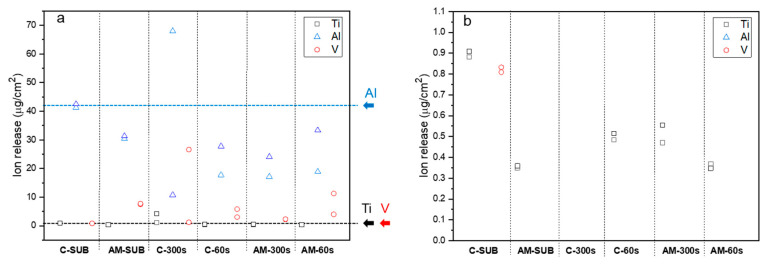
(**a**) Scattered diagram of ion release with respect to the exposed sample area of bare, flash-PEO (30 s)- and PEO (300 s)-coated AM and conventional Ti6Al4V alloys after 30 days of immersion and corrosion testing in SBF. Arrows mark the Ti, Al and V ion release from bare wrought alloy, as a reference. (**b**) Zoomed-in Ti release range.

Metal ion release into SBF from the tested systems after 30 days of corrosion exposure is presented in Figure 15 and Supplementary Table S4.

The AM substrate exhibited lower release of Ti^4+^ (−60.2%) and Al^3+^ (−26.2%) compared with the reference conventional Ti6Al4V substrate, which can be attributed to a more uniform microstructure and the lower β-phase content intrinsic to the DMLS process. Conversely, the release of V^5+^ from the AM substrate was over eight times higher than that from the conventional alloy (7.49 vs. 0.81 µg/cm^2^), which is consistent with the overall greater content of V in AM alloy (Table 1).

In the coated AM group, the 300 s PEO treatment showed better performance than flash-PEO in suppressing the release of Al^3+^ and V^5+^ ions: Al^3+^ release decreased on average by 32.2% and V^5+^ release decreased by 69.6% compared to the substrate. Ti^4+^ release, on the other hand, increased by 45.7%.

The PEO coatings on the conventional substrate exhibited different behavior. In flash-PEO coating, Al^3+^ (−44.8%) and Ti^4+^ (−43.2%) showed significant reductions. In the PEO coating, Ti^4+^ release increased by 203.4%, whereas V^5+^ release rose by 1615%. This massive lixiviation of V species is consistent with the EDS data for the 300 s coating cross-section, which revealed the incorporation of up to 2.6 at.% V (Table 4). The average release of Al^3+^ at 300 s was only marginally lower than in the uncoated condition (−4.5%).

## 4. Discussion

### 4.1. Flash-PEO Rationale

Previously, Mora-Sánchez et al. explored flash-PEO treatments of DMLS AM Ti6Al4V alloy as short as 35 s and 120 s in an electrolyte containing only Ca and P as bioactive species [5]. These coatings were shown to successfully prevent crevice corrosion, which the AM alloy suffered under polarization at ≥1.5 V in α-MEM cell culture medium. For comparison, conventional Ti alloys are known to undergo localized corrosion in saline solutions (such as seawater or physiological solutions) only at ≥70 °C [25]. The fact that AM Ti6Al4V can experience this type of degradation at 37 °C underscores the need for protective coatings.

In contrast, Santos-Coquillat et al. demonstrated that a multi-elemental approach to the design of PEO coatings for commercially pure Grade I Ti supported accelerated bone tissue remodeling in vitro [16]. They applied a 300 s PEO treatment in an electrolyte containing Ca, P, Si, Mg and Zn, which yielded the lowest passive current density while providing a considerable release of all five bioactive elements over 28 days of immersion in SBF.

Considering the above, the main interest of the present work was to investigate the viability of multi-elemental flash-PEO coating in terms of ensuring long-term corrosion protection of DMLS Ti6Al4V alloy and its effect on the release of potentially cytotoxic Al^3+^ and V^5+^ ions. A treatment time of 60 s was selected to ensure that sparking lasted long enough to enable sufficient incorporation of bioactive elements, while also reducing energy consumption by half compared with [5]. Longer, 300 s PEO treatment and the conventionally processed Ti6Al4V alloy were used as a reference.

Our energy-saving approach demonstrated that the incorporation of all bioactive elements in both wrought and AM Ti6Al4V alloys was equal or greater than that obtained by Santos-Coquillat on Grade I Ti alloy. Furthermore, increasing the treatment time did not lead to any additional practical enrichment of the coatings with bioactive elements. Notably, these elements were mostly present in the amorphous phase, whose content in flash-PEO was 16% higher for the AM alloy than for the wrought alloy. This may be attributed to the earlier onset of sparking (and the associated rise in local surface temperature) during the PEO of wrought alloy, which also promoted rutile phase formation. The amorphous state of bioactive species is known to assist their lixiviation [26].

### 4.2. Electrochemical Stability and Protection Mechanism

The revision of the state of the art on corrosion of AM Ti alloys demonstrates that their corrosion behavior is heavily influenced by their manufacturing method and the resulting microstructure. Laser powder bed fusion (LBPF) AM techniques such as DMLS (or SLM) often produce acicular α′ martensite and porosity, which compromise passive film stability and increase susceptibility to corrosion [27,28,29,30]. Post-processing treatments like heat treatment and Hot Isostatic Pressing (HIP) can transform these unstable phases into lamellar α + β or Widmanstätten structures, significantly improving corrosion resistance [29,30,31]. Comparative studies show that heat-treated AM samples exhibit lower corrosion rates and more uniform oxide layers than as-built counterparts [28,31], with HIP-treated Ti6Al4V showing reduced corrosion current density and enhanced passive film stability [31]. In the present work, we adhered to the heat treatment employed in our previous works on DMLS Ti6Al4V alloy.

Surface coatings and alloying strategies further enhance the corrosion resistance and biocompatibility of Ti alloys. Coatings such as hydroxyapatite (HA), Ag-doped dicalcium phosphate dihydrate (DCPD), and polydopamine (PDA) composites with Cu/Mn ions were shown to promote bioactivity and reduce degradation in simulated body fluids [32,33,34,35,36,37]. The 4% Ag-DCPD coating demonstrated superior electrochemical stability and dense HA layer formation [32], while PDA coatings showed biphasic ion release beneficial for antibacterial activity and bone regeneration [34].

Although PEO coatings have demonstrated significant improvements in corrosion resistance, especially when formed in phosphate electrolytes [35], long-term studies on AM titanium alloys remain limited. Most research focuses on short-term electrochemical testing, with few investigations addressing the unique challenges posed by AM microstructures over extended periods [34,35]. Studies on Sr and Zn-doped PEO films suggest potential for tailored surface treatments in biomedical applications [38]. However, the lack of standardized protocols and extended exposure data underscores the need for further research to optimize coating performance and ensure long-term durability in demanding environments such as aerospace and biomedical fields [34,38].

In the present work, we have chosen a 30 days testing in SBF, which closely represents the human blood plasma. Furthermore, one month is a typical post-orthopaedic surgery period during which osseointegration takes place, the operated patient is expected to resume gentle walking and it becomes clear whether the possibility of immediate complications can be discarded.

Initial electrochemical measurements using PDP disclosed that the AM alloy in untreated state was more passive compared to the conventional alloy, whereas the latter was more passive in the treated state. PEO, and particularly flash-PEO coatings, facilitated the oxygen evolution reaction (here written for neutral medium):

4 OH^−^ → 2 O_2_ + 2 H_2_O + 4 e^−^

which occurred at ~0.6–0.8 V earlier compared with bare substrates. This is related to the easier charge transfer through the PEO oxide material and the PEO/substrate interface. This can be attributed to the crystallinity of the coatings and the presence of anatase in them [39], as opposed to amorphous passive films of non-coated substrates.

EIS measurements helped to understand how the protective coatings work when an implant is at its open circuit, i.e., corrosion potential. Typically, for most alloy systems (e.g., steels, aluminium, magnesium), a total impedance modulus is equated to the system’s polarization resistance and, therefore, is an adequate representation of its corrosion resistance. The same cannot be said about Ti alloys, since their passive oxide film is an n-type semiconductor, in which case the EIS detects the response of the space-charge region at low frequencies [40]. The presence of the space-charge region is typically represented by a series arrangement of two elements (R_sc_/CPE_sc_) (R_b_/CPE_b_) [5,41] when analyzing the response of non-coated Ti.

However, the attempt to use that EEC in the present work for bare wrought and AM alloys produced meaningless values, which is why the EEC depicted in Figure 9 had to be used. A possible reason for the suitability of the parallel arrangement of (R_por_/CPE_por_)/(R_b_/CPE_b_) may lie in the different oxidation rates of basal and prismatic planes of α-Ti grains and the even faster oxidation of β-Ti grains. Matykina et al. have shown that α-Ti grains with basal orientation with respect to the sample surface oxidize faster and develop a much thicker, rougher and more porous oxide film than prismatic-oriented grains [23]. Mora-Sánchez et al. showed that at the onset of spark microdischarge the β-Ti grains disclose the thickest and most porous oxide compared with α-Ti grains [5].

In the present work, the alloys were etched in Keller solution for a considerably long time (180 s), which activated the surface of the grains, making them more susceptible to passivation upon immersion and polarization. Hence, it makes sense to assume that the (R_por_/CPE_por_) element represents the porous oxide film formed on β-grains and basal α-Ti grains, whereas (R_b_/CPE_b_) represents the compact film formed on prismatic α-Ti.

**Figure 16:**
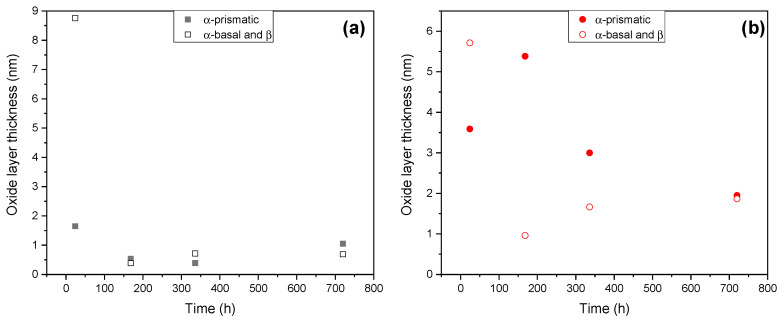
Evolution of the calculated thickness of the passive film on α-basal, α-prismatic and β grains of AM (**a**) and conventional (**b**) substrates.

We tested this idea by using Brug’s formula (C = CPE^1/n^ R^(1−n)/n^) to calculate the true capacitance for the films from the CPE_por_ and CPE_b_ values. The film thickness values were then obtained (d = ε_0_ ε C^−1^), where ε_0_ and ε are, respectively, the permittivity of vacuum, 8.854 × 10^−14^ Fcm^−1^, and the dielectric constant ε = 57, frequently reported for TiO_2_ [20]. Figure 16 shows that the initial difference between the oxide film thickness (8.7 nm vs. 1.6 nm) formed on different grains in AM alloy disappears within first 7 days of immersion and a relatively uniform 0.7–1.0 nm-thick film is formed by the end of testing. In the case of the conventionally processed alloy, film thickness levelling takes a longer time to achieve a ~2 nm film. Importantly, in the long run, the thinner passive film on the AM alloy is less defective and, consequently, exhibits 6 times greater resistance than that of the conventional alloy (Supplementary Table S1).

The coating morphology and microstructure had a notable effect on the corrosion performance of the alloys. The inner laterally connected pores in the flash-PEO coating on the AM alloy were sealed (R_por_ = 1.45 kΩcm^2^), which is possibly related to the pH conditions favorable for precipitation created in these bottlenecked areas. These inner regions are difficult to access for the electrolyte and mass transfer can only occur there by diffusion.

It should also be noted that the resistance of the coating’s barrier layer is not yet manifested at 10^−2^ Hz, given that the observed |Z|_0.01Hz_ values are ~5–10 times lower than the R_b_ values obtained from fittings. For instance, the simulation of the AM-60 s spectrum using fitted EEC parameters down to much lower frequencies demonstrates that a purely resistive response of the barrier layer would be expected at ~10^−10^ Hz (in 317 years).

According to the i_pass_ values obtained from PDP experiments following 1 h of exposure, the protection provided by the coating systems can be ranked in the following order: C-300 s > AM-300 s> C-60 s > AM-60 s. This correlates with the highest R_b_ values (~1.5 and ~1.8 MΩcm^2^) exhibited by the C-300 s at the beginning and at the end of the exposure. However, in the long run the AM-60 s coating is the second best due to the increased stability of its barrier layer (R_b_~0.8 MΩcm^2^) and a drastic increase of the R_por_ over time, favored by the bottlenecked coating microstructure.

The difference in the protective properties of the barrier layer of coated AM and wrought systems can be related to the alloy microstructure as follows. The barrier layer of a PEO coating is formed at the last stage of each microdischarge event’s life. When plasma is extinguished, the material cools and oxidation proceeds according to conventional anodizing. It is known that barrier layer growth and defect formation depend on the alloy microstructure, i.e., on the phase, grain size and orientation. Further, the initiation of dielectric breakdown events and their propagation also depend on the aforementioned characteristics. We have previously shown that microdischarge events spread faster and more uniformly over the DMLS Ti6Al4V alloy than over the wrought alloy [5]. In the latter, some α grains remain largely unaffected by dielectric breakdown even after 25 s of treatment.

Unlike the coarse-grained equiaxial microstructure of the wrought mill-annealed alloy, the AM alloy’s lamellar microstructure is finer, and its grain size is closer to the size of an individual microdischarge event. The width of α-lamellae in the DMLS alloy is about 1 μm, and the size of β grains is far less than that (Figure 13d). Also, the distance between adjacent β grains is much smaller (<1 μm) in the AM alloy than in the wrought alloy (5–10 μm). According to the consensus on PEO, the size of an individual plasma microdischarge is ≤1μm. It can be larger if a cascade of microdischarge events occurs in close proximity to each other, i.e., as a cluster [42]. As a result, the heat-affected zones of microdischarge events at two adjacent β grains of the AM alloy will overlap and span across an α grain between them, resulting in a more uniform temperature distribution and uniform coating growth across the surface. This, in turn, leads to greater homogeneity of the barrier layer and a wider space-charge region in the AM alloy, as evident from the R_b_, R_sc_ and CPE_sc_ values of AM-60 s system (Figure 12).

### 4.3. Metal Ion Release

The ICP-OES measurements were performed to correlate ion release with the corrosion rate, determine the chemical stability of the coatings and provide an outlook for their potential cytotoxicity. A large difference between the amounts of oxidized Ti over 30 days, based on the i_corr_ values, and Ti^4+^ detected in the solution by ICP-OES (Table 8), indicates that a significant portion of Ti^4+^ is incorporated into the passive film. When converted into the resultant TiO_2_ thickness, it shows excellent agreement with the passive layer thickness calculated from EIS data analysis for bare substrates. It should be borne in mind that, for simplicity, all our calculations assumed pure TiO_2_, while in reality Al^3+^ and V^5+^ are incorporated into the oxide film.

Considering Ti^4+^ release from the coatings, it is 1–2 orders of magnitude lower than expected from the respective i_corr_ values (Table 5), but comparable to or even somewhat higher than the release measured for the respective bare substrates (Figure 15). This means that the coatings, not the substrates, are the main source of metal ion release, undoubtedly due to the large fraction (~50%) of amorphous TiO_2_ and the large real surface area resulting from porosity.

As for the V^5+^ ion release, in all cases it was similar to or greater from the coatings than from the bare substrates. This trend is in agreement with the previous findings of Matykina et al. [18] for 90 s PEO coatings on conventional Ti6Al4V alloy exposed to SBF for up to 8 weeks. Conversely, in this work, the 60 s PEO treatments were beneficial for suppressing Al^3+^ release (by ~32–45%), because the incorporation of substrate species into the coating directly depends on the duration and intensity of the microdischarges. Of all the coatings, it appears that the bottleneck pore morphology of the AM alloy in particular facilitated the release of Al^3+^ and V^5+^ ion release from the coatings (Figure 15), due to the easy access of the electrolyte through the continuous inner cavities to the barrier layer, where the content of these elements is higher.

Overall, the accumulated levels of both cytotoxic species were considerably above their normal levels in blood (30 ppb for V and 3 ppb for Al ions [43,44,45]). Al ion cytotoxicity with respect to mouse mesenchymal stem cells is observed from 1 ppm [46]. V ion cytotoxicity with respect to mouse fibroblasts is expressed at 23 μM, or 1173 ppb [47]. The 30- day accumulated values from flash-PEO coating on DMLS AM alloy were ~260 ppb and ~913 ppb for V and Al, respectively, in ~100 mL of tested SBF. For the flash-PEO on wrought alloy these values were, respectively, 3 and 29% lower. If an implant of the same surface area as the samples in this study (4 cm^2^) were in a body (i.e., in ~5 L of blood), and assuming that the ions were not effectively excreted through kidneys due to some health conditions, then these concentrations would be, respectively, 5.2 ppb and 18.3 ppb, i.e., within the norm for V but way above it for Al. Assuming no metal accumulation in a healthy individual, the daily release of Al (26 μgcm^−2^ d^−1^) and V (0.25 μgcm^−2^ d^−1^) would amount to ~0.6 ppb and ~0.2 ppb, both of which are safe levels. A flash-PEO-coated DMLS implant of 20 cm^2^ surface area (e.g., a small plate) would generate 3 ppb of Al in blood. An implant of ~7000 cm^2^ surface area would be needed to reach the 1 ppm cytotoxicity threshold of Al. For comparison, the surface area of a human adult femur is 350–400 cm^2^. That said, in vitro cytotoxicity tests with different cell types are pending for future work.

The potential incompatibility of the DMLS Ti6Al4V alloy with kidney-compromised patients emphasizes the need for its replacement with β-type alloys like Ti-42Nb, Ti-13Nb-13Zr and Ti-12Cr-3Sn, enriched with Nb, Zr, Ta, Cr, and Sn, which form stable oxide layers that resist chloride attack and minimize toxic ion release [35,36,37,48].

## 5. Conclusions

The DMLS Ti6Al4V alloy demonstrates superior long-term passive behavior, forming a film that is twice as thin but 6 times more resistant than that of a wrought Ti6Al4V reference. The non-coated alloy releases ~60% less Ti and ~25% less Al than its wrought counterpart, but yields ~900% more V ion release.

The findings highlight the crucial role of the flash-PEO coating morphology in controlling the corrosion behavior of AM Ti6Al4V in biological environments. The energy-efficient flash-PEO treatment, as short as 60 s, successfully enriches the coating material with Ca, P, Si, Mg and Zn, while providing high barrier layer resistance.

The flash-PEO coating is the main source of metal ion release, but it is effective in maintaining the release of cytotoxic Al and V ions from the DMLS alloy system within normal daily levels, provided there is effective kidney function in a healthy individual.

## Figures and Tables

**Table 1:** Local EDS analysis (at.%) of conventional (C) and DMLS (AM) substrates after etching in Keller solution, as per locations in Figure 1.

Specimen/ Location		Na	Mg	Al	Si	P	S	K	Ti	Ca	Zn	O	V	Fe
C	Area	-	-	9.6	-	-	-	-	87.3	-	-	-	3.1	-
AM	1	-	-	8.2	-	-	-	-	92.1	-	-	-	5.6	0.7
	2	-	-	6.6	0.09	-	-	-	93.5	-	-	-	3.8	0.1
	3	-	-	7.3	-	-	-	-	91.8	-	-	-	5.8	0.8

**Table 2:** Local EDS analysis (at.%) of conventional (C) and DMLS (AM) substrates with flash-PEO (60 s) and PEO (300 s) coatings, as per locations in Figure 3.

Specimen/ Location		Na	Mg	Al	Si	P	S	K	Ti	Ca	Zn	O	V	Fe
AM-300 s (SS)	Plan view area	0.8	2.5	0.7	14.1	4.1	1.0	0.2	5.5	1	-	70.2	-	-
C-300 s (SS)		-	-	0.5	11.6	3.7	4.6	-	3.4	5.6	-	70.6	-	-
AM-300 s		0.8	2.5	0.7	13.8	4.1	0.9	0.2	5.4	1	-	70.4	0.2	-
AM-60 s		-	1.3	1.7	3.5	7.5	0.3	-	26.7	9.4	0.9	47.5	1.3	-
C-300 s		-	1.1	1.8	2.7	5.2	0.3	-	16.2	2.0	1.1	69.0	0.8	-
C-60 s		0.9	1.3	1.4	3.7	8	0.2	-	25.4	10	1	46.9	1.2	-

**Table 5:** Corrosion and passive current densities following 1 h of immersion in SBF.

Specimen	E_corr_ (V_Ag/AgCl_)	i_corr_ (µA/cm^2^)	i_pass_ (µA/cm^2^)
AM-Sub	−0.185	0.05	2.0
C-Sub	−0.244	0.17	3.8
AM-PEO 60 s	0.130	0.16	0.66
AM-PEO 300 s	0.258	0.09	0.47
C-PEO 60 s	0.268	0.19	0.47
C-PEO 300 s	0.221	0.08	0.094

**Table 6:** Local EDS analysis (at.%) of conventional (C) and DMLS (AM) substrates with flash-PEO (60 s) and PEO (300 s) coatings, after 30 days immersion in SBF as per Figure 13 and Figure 14.

Specimen/ Location		Na	Mg	Al	Si	P	K	Ti	Ca	Zn	O	V
C-sub	1	-	-	7.7	-	-	-	70.9	-	-	18.6	2.8
AM-sub	1	-	-	8.8	-	-	-	71.1	-	-	17.9	2.2
	2	-	-	9.9	-	-	-	75.4	-	-	12.1	2.7
AM-300 s	1	1.9	0.9	1.2	5.3	8.5	-	16.1	9.0	0.6	55.9	0.8
	2	-	0.7	1.7	4.0	6.3	-	17.2	5.4	0.5	63.5	0.7
C-300 s	1	-	1.1	1.5	3.5	6.5	-	16.5	6.5	0.4	63.5	0.5
	2	-	1.1	1.3	3.4	5.7	0.2	17.6	6.5	0.5	62.9	0.7
AM-60 s	Plan view area	-	1.4	1.1	3.2	6.6	-	11.1	6.5	0.4	69.2	0.5
C-60 s		0.7	1.1	1.2	2.9	5.7	-	13.2	5.5	0.4	68.6	0.7

**Table 7:** Roughness parameters and water contact angle before and after 30 days of immersion in SBF and corrosion testing.

Sample		WCA, °	S_a_, μm	S_10z_, μm
AM-Sub	before	73.9	0.12 ± 0.01	1.0 ± 0.1
	after	67.7	0.63 ± 0.04	4.9 ± 1.1
C-Sub	before	61.8	1.47 ± 0.11	11.1 ± 1.3
	after	49.9	0.2 ± 0.03	5.0 ± 2.3
AM-60 s	before	57.3	0.32 ± 0.02	4.7 ± 1.7
	after	62.4	1.2 ± 0.4	9.7 ± 5.4
AM-300 s	before	58.3	0.53 ± 0.04	6.5 ± 3.8
	after	49.1	1.3 ± 0.36	11.7 ± 5.2
C-60 s	before	61.8	0.35 ± 0.03	3.8 ± 0.5
	after	68.4	0.54 ± 0.2	4.5 ± 0.8
C-300 s	before	62.5	0.55 ± 0.04	5.1 ± 0.7
	after	64.8	1.32 ± 0.52	9.7 ± 3.8

**Table 3:** Pore data estimated from image analysis of SEM micrographs.

Specimen	Pore Population Density, mm^−2^	Average Pore Size, µm	Porosity, %
AM-60 s	1.32 × 10^6^	0.8	9.7
AM-300 s	0.29 × 10^6^	3.4	14.1
C-60 s	0.90 × 10^6^	1.1	13.1
C-300 s	0.56 × 10^6^	2.8	19.8

**Table 4:** Local EDS analysis (at.%) of cross sections of conventional (C) and DMLS (AM) substrates with flash-PEO (60 s) and PEO (300 s) coatings, as per locations in Figure 4.

Specimen/ Location			Na	Mg	Al	Si	P	S	K	Ti	Ca	Zn	O	V	Fe
AM-300 s	Cross-sections	1	-	-	2.5	0.2	0.3	-	-	22.8	0.1	0.1	71.6	-	-
		2	0.7	0.4	1.6	2.5	5.5	1.3	0.3	17.9	2.1	0.2	66.8	0.7	-
		3	0.5	0.6	2.1	2.4	4.2	-	-	14.0	2.7	0.2	0.5	-	-
AM-60 s		1	0.3	0.4	2.6	1.3	2.9	0.7	-	19.9	1.3	0.7	69.8	0.7	-
		2	0.2	1.2	1.8	1.8	5.2	0.2	-	11.2	4.4	3.3	72.8	0.4	-
C-300 s		1	1.4	-	2.5	0.3	0.8	2.6	0.1	27.7	-	-	62.0	2.6	-
		2	1.1	0.4	1.3	1.6	4.8	1.4	0.2	17.2	0.9	0.4	68.5	2.1	-
		3	-	1.3	1.4	3.6	4.7	0.2	-	11.4	1.8	0.8	73.5	1.4	-
C-60 s		1	0.4	-	2.8	1.0	3.4	1.5	-	22.5	0.4	0.1	67.3	0.6	-
		2	0.6	1.5	1.4	3.5	5.8	-	-	11.0	7.3	0.2	68.0	0.7	-

**Table 8:** Comparison of the PDP, ICP-OES and EIS derived values for oxidized Ti, Ti^4+^ lost into the SBF and barrier TiO_2_ thickness over 30 days of immersion in SBF.

Specimen	Oxidized Ti, μg cm^−2^	Ti^4+^ in SBF, μg cm^−2^	Ti^4+^ Incorporated into the Passive Film, μg cm^−2^	TiO_2_ Thickness Gain, nm	TiO_2_ Thickness from EIS, nm
C-SUB	54.65	0.88	53.77	2.34	2
AM-SUB	16.07	0.35	15.72	0.68	0.7
C-300 s	25.72	2.67	23.05	1.00	-
C-60 s	61.08	0.50	60.58	2.63	-
AM-300 s	28.93	0.51	28.42	1.23	-
AM-60 s	51.43	0.36	51.07	2.22	-

## Data Availability

All data presented in this work will be made available through Docta Complutense repository. https://docta-ucm-es.bucm.idm.oclc.org/handle/20.500.14352/16.

## Supplementary Materials

The following supporting information can be downloaded at https://www.mdpi.com/article/10.3390/jfb16100362/s1, Table S1. Fitted EEC parameters for EIS spectra of substrates; Table S2. Fitted EEC parameters for EIS spectra of flash-PEO-coated specimens; Table S3. Fitted EEC parameters for EIS spectra of PEO-coated specimens; Table S4: Average values of ion release from bare and coated AM and conventional Ti6Al4V alloys after 30 days of immersion and corrosion testing in SBF; Figure S1: Macrographs of the PEO treated Ti6Al4V alloy samples (300 s) showing the extent of the sample area affected by soft sparking; Figure S2: 2D topographical mapping obtained by optical profilometry of the bare and coated alloys before and after 30 days of immersion in SBF and corrosion testing: (a,b) bare substrates, (c,d) flash-PEO-coated substrates, (e,f) PEO-coated substrates.

## Abbreviations

The following abbreviations are used in this manuscript:

AM	Additive Manufacturing
CPE	Constant Phase Element
DCPD	Doped dicalcium phosphate dihydrate
EDS	Energy Dispersive Spectroscopy
EIS	Electrochemical Impedance Spectroscopy
HA	Hydroxyapatite
HIP	Hot Isostatic Pressing
ICP-OES	Inductively Coupled Plasma Optical Emission Spectroscopy
LBPF	Laser Powder Bed Fusion
OCP	Open Circuit Potential
PDA	Polydopamine
PDP	Potentiodynamic Polarization
PEO	Plasma Electrolytic Oxidation
SBF	Simulated Body Fluid
SEM	Scanning Electron Microscopy
SLM	Selective Laser Melting
WCA	Water Contact Angle
XRD	X-Ray Diffraction
