# Supplementary material for: Metal Ion Release from PEO-Coated Ti6Al4V DMLS Alloy for Orthopedic Implants
Source: J Funct Biomater. 2025 Sep 28;16(10):362. doi: 10.3390/jfb16100362 (PMC12565525; doi:10.3390/jfb16100362)
Supplement: Supplementary file 1 [file jfb-16-00362-s001.zip › jfb-3859301-supplementary.pdf]

Supplementary Table S1. Fitted EEC parameters for EIS spectra of substrates.

Substrate	Time	CPE_{por}, $\mu Ss^n cm^{-2}$	n_{por}	R_{por}, $k\Omega cm^2$	D_{por}, nm	CPE_b, $\mu Ss^n cm^{-2}$	n_b	R_b, $k\Omega cm^2$	D_b, nm	$\lvert Z\rvert_{0.01Hz}$, $k\Omega cm^2$
AM Ti6Al4V	1 h	17.23	0.87	0.038	8.76	13.37	0.85	4135	1.65	768
	7 d	50.33	0.77	4.81	0.39	15.20	0.60	1699	0.54	171
	14 d	50.10	0.79	72.17	0.72	15.99	0.59	2085	0.39	120
	30 d	47.89	0.80	110.30	0.69	19.40	0.70	987	1.05	134
Conv. Ti6Al4V	1 h	18.34	0.91	0.034	5.71	9.96	0.90	2209	3.59	432
	7 d	21.00	0.83	415.76	0.96	3.28	0.76	8489	5.38	410
	14 d	26.71	0.86	79.35	1.67	12.26	0.57	124	3.00	140
	30 d	26.47	0.84	42.04	1.87	12.87	0.52	165	1.95	130

Supplementary Table S2. Fitted EEC parameters for EIS spectra of flash-PEO coated specimens.

PEO 60s	Time	CPE_{port}, $\mu Ss^n cm^{-2}$	n_{por}	R_{por}, $k\Omega cm^2$	CPE_{sc}, $\mu Ss^n cm^{-2}$	n_{sc}	R_{sc}, $k\Omega cm^2$	CPE_b, $\mu Ss^n cm^{-2}$	n_b	R_b, $k\Omega cm^2$	$\lvert Z\rvert_{0.01Hz}$, $k\Omega cm^2$
AM Ti6Al4V	1 h	9.84	0.62	0.025	18.65	0.76	1.51	18.77	0.83	403	192.5
	7 d	2.33	0.76	8.11	17.99	0.39	61.6	107.3	0.58	2036	90.9
	30 d	3.89	0.73	1.45	22.2	0.42	15.9	43.78	0.33	817	66.9
Conv. Ti6Al4V	1 h	30.80	0.53	0.525	2.34	0.85	9.48	5.26	0.85	871	192.5
	7 d	9.83	0.74	0.035	0.21	0.96	1.08	74.9	0.45	1092	42.6
	30 d	5.99	0.77	0.017	1.55	0.82	0.92	80.9	0.43	782	38.2

Supplementary Table S3. Fitted EEC parameters for EIS spectra of PEO coated specimens.

PEO 300s	Time	CPE_{por}, $\mu Ss^n cm^{-2}$#	n_{por}#	R_{por}, $k\Omega cm^2$#	CPE_{sc}, $\mu Ss^n cm^{-2}$#	n_{sc}#	R_{sc}, $k\Omega cm^2$#	CPE_b, $\mu Ss^n cm^{-2}$#	n_b#	R_b, $k\Omega cm^2$#	$\lvert Z\rvert_{0.01Hz}$, $k\Omega cm^2$ #
AM Ti6Al4V	1 h	6.73	0.68	0.095	8.82	0.82	1.60	29.50	0.61	499	125.6
	7 d	0.51	0.83	0.074	6.24	0.60	4.07	42.19	0.58	391	97.7
	30 d	0.39	0.81	0.351	8.28	0.54	4.76	45.69	0.57	303	86.8
Conv. Ti6Al4V	1 h	0.47	0.84	0.066	7.07	0.92	4.09	6.33	0.66	1562	671
	7 d	18.09	0.58	0.022	0.76	0.99	4.75	11.27	0.75	533	154.2
	30 d	0.62	0.74	2.320	5.11	0.57	7.63	13.20	0.45	1849	235.67

Supplementary Table S4. Average values of ion release from bare and coated AM and conventional Ti6Al4V alloys after 30 d of immersion and corrosion testing in SBF.

Specimen	Ti^{4+}, μg cm^{-2}	Al^{3+}, μg cm^{-2}	V^{5+}, μg cm^{-2}
C-SUB	0.88	41.26	0.81
AM-SUB	0.35	30.41	7.49
C-300s	2.67	39.36	13.89
C-60s	0.50	22.75	4.42
AM-300s	0.51	20.61	2.28
AM-60s	0.36	26.09	7.64

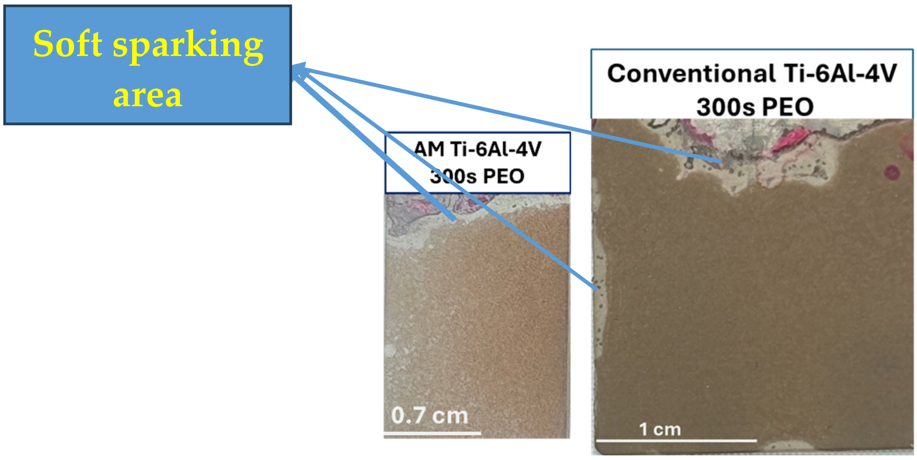

Supplementary Figure S1. Macrographs of the PEO treated Ti6Al4V alloy samples (300 s) showing the extent of the sample area affected by soft sparking.

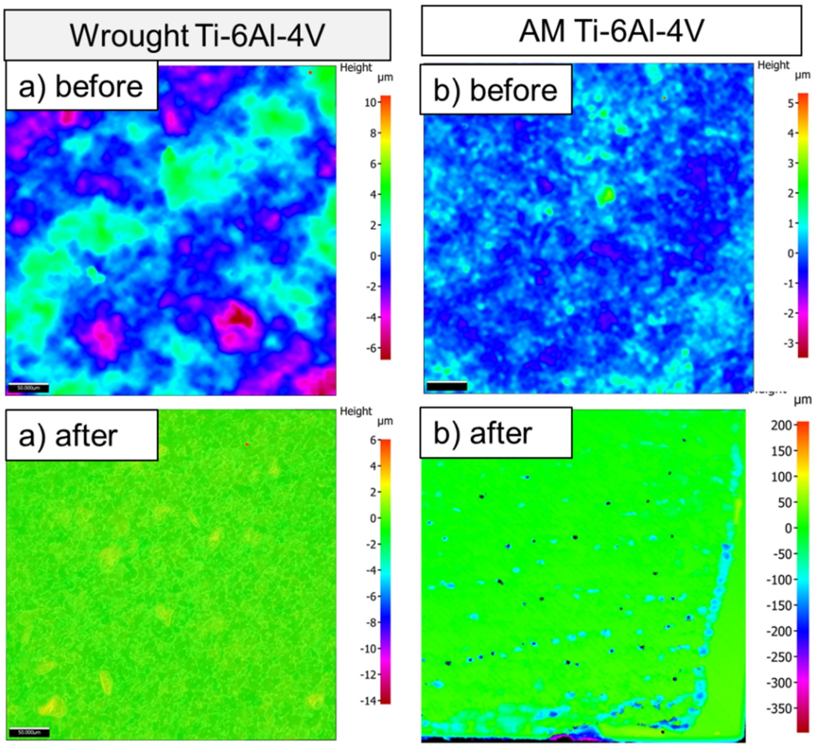
Wrought Ti-6Al-4V
AM Ti-6Al-4V
a) before
b) before
a) after
b) after
Height
µm

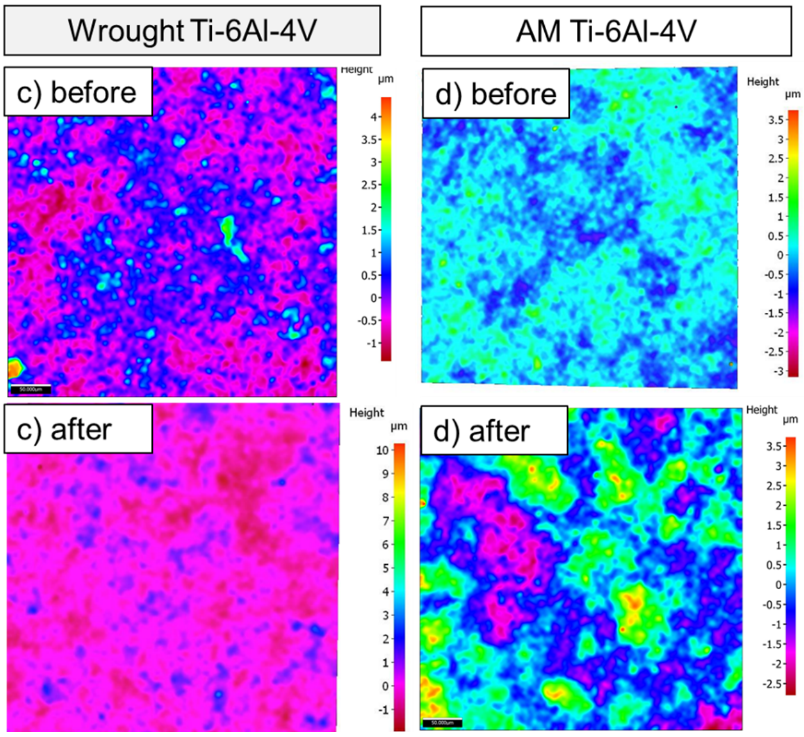
Wrought Ti-6Al-4V
AM Ti-6Al-4V
c) before
d) before
c) after
d) after
Height
µm

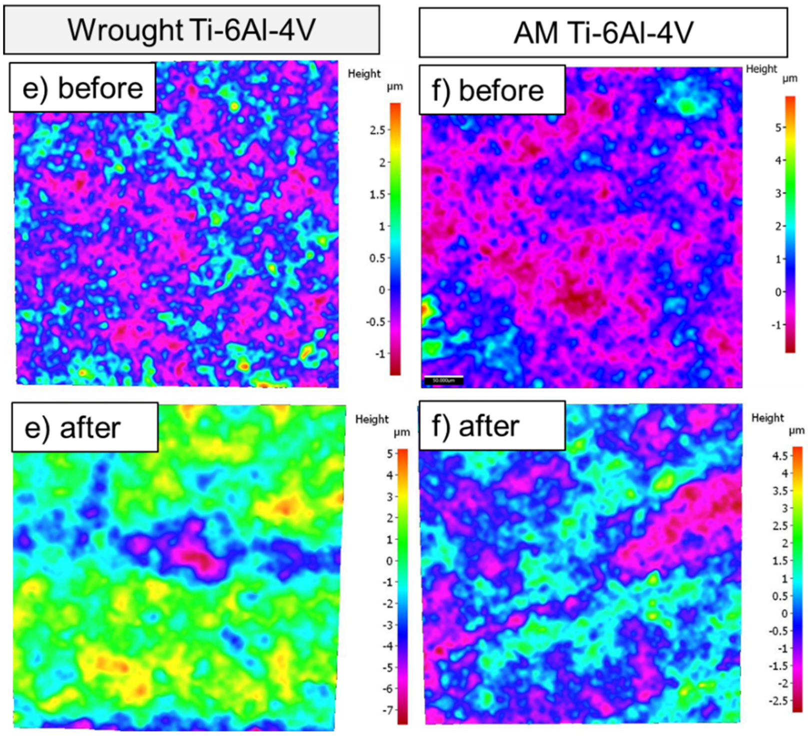

Supplementary Figure S2. 2D topographical mapping obtained by optical profilometry of the bare and coated alloys before and after 30 d of immersion in SBF and corrosion testing: **a, b**) bare substrates, **c, d**) flash-PEO coated substrates, **e, f**) PEO-coated substrates.
